# Applying language models for suicide prevention: evaluating news article adherence to WHO reporting guidelines

**DOI:** 10.1038/s44184-025-00139-5

**Published:** 2025-06-20

**Authors:** Zohar Elyoseph, Inbar Levkovich, Eyal Rabin, Gal Shemo, Tal Szpiler, Dorit Hadar Shoval, Yossi Levi Belz

**Affiliations:** 1 School of Counseling and Human Development, The Faculty of Education, The University of Haifa, Haifa, Israel; 2 Department of Brain Science, Faculty of Medicine, Imperial College London, London, UK; 3 The Center for Psychobiological Research, Department of Psychology, Max Stern Yezreel Valley College, Tel Adashim, Israel; 4 Faculty of Education, Tel Hai College, Upper Galilee, Israel; 5 Department of Psychology and Education, the Open University of Israel, Ra’anana, Israel; 6 Department of Psychology, The Center for Psychobiological Research, Max Stern Yezreel Valley College, Yezreel Valley, Israel; 7 The Lior Tsfaty Center for Suicide and Mental Pain Studies, Ruppin Academic Center, Emek Hefer, Ruppin, Israel

**Keywords:** Psychology, Health care, Risk factors

## Abstract

The responsible reporting of suicide in media is crucial for public health, as irresponsible coverage can potentially promote suicidal behaviors. This study examined the capability of generative artificial intelligence, specifically large language models, to evaluate news articles on suicide according to World Health Organization (WHO) guidelines, potentially offering a scalable solution to this critical issue. The research compared assessments of 40 suicide-related articles by two human reviewers and two large language models (ChatGPT-4 and Claude Opus). Results showed strong agreement between ChatGPT-4 and human reviewers (ICC = 0.81–0.87), with no significant differences in overall evaluations. Claude Opus demonstrated good agreement with human reviewers (ICC = 0.73–0.78) but tended to estimate lower compliance. These findings suggest large language models’ potential in promoting responsible suicide reporting, with significant implications for public health. The technology could provide immediate feedback to journalists, encouraging adherence to best practices and potentially transforming public narratives around suicide.

## Introduction

As artificial intelligence (AI) continues to advance, large language models (LLMs) such as Google’s Gemini, Claude.AI, and OpenAI’s ChatGPT are showcasing capabilities that hold great promise for mental healthcare^1–4^. These technologies have the potential to revolutionize the field by accelerating research, providing valuable assistance to clinicians, and offering support to patients^5–7^. Despite these promising capabilities, the practical application of LLMs in directly contributing to mental health improvements, particularly in public health initiatives such as suicide prevention, remains underexplored^8,9^. The aim of the current study was to bridge this gap by investigating how LLMs can be effectively utilized in the global fight against suicide, focusing on the critical role of media and journalism in mental health advocacy and awareness.

How to prevent the next suicide is one of the most common and still highly complicated questions in the field of psychology and psychiatry. Indeed, it is a question of critical importance given that suicide poses a grave public health concern, with ~817,000 individuals dying by suicide annually^10^. This figure underscores the urgent necessity for a comprehensive investment in a broad array of suicide prevention strategies aimed at curtailing the global suicide prevalence. Among other main targets, such as treating depression and reducing access to lethal means, media coverage of suicide is known to be an important target that may reduce the global suicide risk^11^. Media portrayals of suicides have been scrutinized for their potential influence on suicide rates, with numerous studies revealing a link between such coverage and subsequent increases in suicide occurrence^6,12–15^. The “copycat effect”—the propensity of vulnerable individuals to mimic suicidal acts depicted in the media—exemplifies the risks associated with detailed and sensationalized reporting of suicide methods^13,16,17^. For example, reporting of deaths of celebrities by suicide appears to have had a meaningful impact on total suicides in the general population, which mostly uses the same suicide method as described in the articles^11^. On the other hand, it is important to note the beneficial impact of responsible media coverage, known as the “Papageno effect,” which frames suicide as preventable and offers information on prevention and intervention, thereby encouraging individuals in crisis to seek help^11,18^. For example, media narratives of hope and recovery from suicidal crises appear to have a beneficial effect on suicidal ideation among at-risk individuals^19^.

Taken together, several systematic reviews have emphasized that the ways in which media covers suicide stories have an important contribution to suicide rates (e.g., ref. ^13^).

The challenge of ethical media coverage extends beyond suicide reporting to various sensitive topics, necessitating broader content-moderation strategies. Previous studies have explored automated systems for detecting and moderating potentially harmful content across various platforms. For instance, studies have examined machine-learning approaches for identifying hate speech and cyberbullying on social media^20^. In the context of mental health, automated tools have been developed to detect signs of depression and anxiety on social media posts ^21–23^ However, the application of such technologies to news article analysis, particularly for sensitive topics, such as suicide, remains relatively unexplored. While some newsrooms employ human editors and fact checkers to ensure ethical reporting, the volume of digital content poses challenges for manual review. This gap underscores the potential of AI-assisted tools to support ethical journalism practices, particularly in areas where human oversight may be limited or inconsistent^24^. This addition provides context for existing technical solutions for content moderation, particularly in the realm of mental health and social media, while also highlighting the gap in applying such technologies to news article analysis. It sets the stage for the study’s focus on using AI to assess adherence to WHO guidelines for suicide reporting.

Following the dual impact of media coverage on suicide rates, the World Health Organization (WHO) formulated guidelines aimed at encouraging responsible reporting. These guidelines advocate for a nuanced approach to suicide reporting, emphasizing the need to avoid sensationalism, reduce the prominence of suicide in news coverage, and provide information on suicide prevention and intervention^25^. By categorizing recommendations into actionable dos and don’ts for journalists, the WHO aims to minimize the risk of imitative suicides while maximizing the potential for media to play a constructive role in suicide prevention. Subjects to be avoided include (1) referencing suicide in the primary headline, (2) disclosing the societal position or significant life occurrences of the individual who has died by suicide, and (3) utilizing unsuitable imagery^25^. Additionally, there exist four supplementary recommendations aimed at encouraging journalists to undertake specific actions, such as disseminating information about available support and intervention mechanisms. An analysis of the extent to which these recommendations (Do’s and Don’ts) are followed will offer deeper insights into the obstacles faced in the application of these guidelines. This evaluation could further inform the focus of training sessions for journalists, emphasizing the concrete steps necessary to comply with these directives. Guidelines for reporting suicide incidents can be grouped into four key dimensions: Prominence, Complexity, Sensationalism, and Prevention. The prominence dimensions (e.g., refrain from explicitly mentioning suicide in the headline), complexity dimensions (e.g., avoiding attributing suicide to a single cause), sensationalism dimensions (e.g., avoiding romanticizing or glorifying the act of suicide), and prevention dimensions (e.g., including information on suicide risk factors and prevention resources)^25^.

Several studies have already shown that while the guidelines are known, adherence to recommended guidelines in suicide-related news stories is only partially followed in different countries and cultures^26–31^. For example, a study examining adherence to recommendation guidelines among suicide stories in Israeli media revealed only partial adherence to established reporting guidelines^32^, with ~49.35% of the articles following recommended practices. This finding indicates significant inconsistencies across and within various criteria. Notably, in addressing the complexity of suicide, the majority of the articles reviewed by Levi-Belz et al.^32^ erroneously attributed the cause to a single factor, contravening the guidelines’ advice against such oversimplification. Furthermore, the analysis highlighted the underrepresentation of mental health issues in suicide narratives^11^. Even after the implementation of special training for journalists, the improvement in adherence was somewhat small and only in part of the guidelines^28,32,33^. Thus, it can be seen that there is a gap in applying and translating scientific knowledge effectively to the Internet and news reports, and that this gap is crucial important to address in preventing future suicides. In the current study, we aimed to take a step forward to narrow this gap, by examined the ability of LLMs to evaluate these criteria, using two LLMs, GPT-4 and Claude.AI. If AI is found effective in monitoring suicide reporting, it could lead to improved automated tools. It can provide timely, multilingual analysis, possibly incorporating clinical insights. Their efficiency allows for ongoing monitoring across various online platforms, including automatic screening of news articles for guideline compliance. This approach may help address some limitations of manual monitoring, offering an alternative method to assess media coverage of suicide.

The aim of the current research was to answer two research questions (RQs):

RQ1: Is the level of agreement between human raters and GenAI raters similar for the overall score of the test and its four dimensions (Prominence, Complexity, Sensationalism and Prevention)?

RQ2: Are there statistically significant differences in the score of the overall test and its four dimensions (Prominence, Complexity, Sensationalism and Prevention) between the four raters (i.e., the human raters and the AI raters)?

If GenAI is found to be effective in this task, it could pave the way for the development of automated tools to assist in monitoring and promoting responsible reporting on suicide.

## Methods

### Data Collection

The Article Selection Process Including this step:

1. Database: Digital archives of ‘Israel Hayom’ and ‘Yedioth Ahronoth’ (2012–2023), which are the two most widely read papers in Israel. It is important to note that both of these newspapers are published in Hebrew, the official language of Israel.
2. Search Parameters: Using Hebrew keywords for “suicide” (in the masculine, feminine, and plural forms), “self-destructive behavior,” “attempted suicide,” and “ended his/her life.
3. Initial Screening: Exclusion of articles focused on suicide bombings or using suicide terminology metaphorically.
4. Dual Review: Independent assessment by two psychology students (undergraduate and graduate) to verify central focus on suicide.
5. Inclusion Criteria: Articles primarily discussing suicide or suicidal behavior.
6. Exclusion Criteria: Articles mentioning suicide peripherally or as a secondary topic.
7. Final Sample: From the initial search results, 15 articles were excluded based on our criteria, resulting in a final sample of 40 articles that met all inclusion requirements.

The sample size was determined using G*Power software, assuming a minimum correlation of 0.8 between raters^32^, a confidence level of 0.80, and an alpha level of 0.05. The results of the analysis indicated the need for a sample size of 40 articles (For access to the articles, see Supplementary table).

#### Article screening criteria

The screening of articles was guided by criteria established by the WHO, as detailed in a study by Levi-Belz et al.^32^ which outlined 15 parameters for article screening. The criteria used are listed in Table 1. Two items (items number 2 and 8) pertaining to the presence of images in articles were excluded from consideration, given the current limitations in analyzing image content^34^. While models like GPT-4 and Claude have made progress in handling visual data, challenges remain in processing fine details, distinguishing between primary and secondary elements, and integrating text and images effectively. Additionally, ethical concerns arise when dealing with sensitive imagery, such as content related to suicide. The questionnaire’s items are divided into four dimensions: prominence (e.g., avoiding explicit mention of suicide in the headline, 2 items), complexity (e.g., avoiding speculation about a single cause of suicide, 3 items), sensationalism (e.g., avoiding glorifying the suicidal act, 5 items), and prevention (e.g., providing information about risk factors for suicide, 3 items)^32^. Each criterion was assessed based on whether it was met.

**Table 1:** WHO guidelines (no. of criterion) for responsible coverage of suicide in the press

(1) Is there any mention of suicide in the headline?
(2) Does the article appear on the front page?
(3) Was the suicidal person described as a celebrity?
(4) Does the article report a single reason for the suicide or suicidal behavior?
(5) Does the article insinuate a connection between a specific life event and suicide or suicidal behavior?
(6) Does the article insinuate a connection between social status and suicide or suicidal behavior?
(7) Does the article insinuate a connection between mental state and suicide or suicidal behavior?
(8) Does the article include inappropriate images?
(9) Does the article present any myths regarding suicide or suicidal behavior?
(10) Does the story include glorifying descriptions of the suicide or suicidal behavior?
(11) Is the suicide or suicidal behavior’s method described in detail?
(12) Is the story descriptive of the location of the suicide or suicidal behavior?
(13) Does the story inform the reader about suicide warning signs/risk factors?
(14) Does the story include any information about prevention?
(15) Does the story include any information about intervention?

### Large language models

The selection of models for this study was based on several key criteria. ChatGPT-4 and Claude.AI were chosen due to their adherence to the following standards: high capabilities in processing the Hebrew language, accessibility to the general public, and their status as leading and high-quality models in their field. These two models represent an advanced version available for a fee, and they are considered the most complex and widely used among professional users and researchers. It’s important to note that the models differ from each other in the databases they were trained on, their unique alignment processes which allows for an interesting comparison of their performance in the specific task of evaluating suicide-related articles.

### ChatGPT4

The aim of ChatGPT-4, created by OpenAI, was to improve upon previous versions in areas such as safety, accuracy, and content policy adherence (OpenAI, 2023). Relative to ChatGPT-3.5, OpenAI has claimed that this latest model (ChatGPT-4) demonstrates an over 82% decrease in harmful or disallowed responses, particularly in areas such as self-harm, illegal activities, and other sensitive topics and a 29% improvement in following safety protocols. This reduction was achieved by incorporating reinforcement learning with human feedback (RLHF) and adversarial testing (OpenAI, 2023). Furthermore, OpenAI reported a 40% improvement in factual correctness over GPT-3.5 (OpenAI, 2023).

As part of the training process, OpenAI solicited abundant feedback from early users and collaborated with over 50 AI ethics experts. However, full details on the architecture changes and training data incorporated have not been disclosed publicly. Without access to such technical specifics, independent auditing remains limited regarding ChatGPT-4’s capabilities. This model currently costs $20 per month.

In the present study we built a specific Custom GPT (https://chat.openai.com/g/g-hOhI4Vgrt-responsible-coverage-ai) named “Responsible Coverage.AI,” using a service called GPTs to create it. GPTs is a service that allows users to add a custom context that will be added to any request of GPT4\ChatGPT. Creating this GPT allowed us to automate the process of evaluating articles.

### Claude Opus

Claude.AI, created by Anthropic, targets beneficial, inoffensive, and truthful output using a constitutional approach (This method trains AI systems by giving them a set of ethical principles or a ‘constitution’ to guide their responses, aims to reduce harm while maintaining helpfulness; Anthropic, 2023). Its 12+ billion parameter transformer model aims to ethically tackle linguistic complexity. The training emphasizes educational data curation and the alignment of model actions with human values and safety considerations, potentially enhancing reliability. A paid Claude.AI subscription recently launched at $20 monthly. In the current study we used the Opus (20$) version.

### Prompt

The following prompt was used to analyze the 40 articles:

*You are acting as a professor of psychology and communications and also as a newspaper editor*.

*Your job is to read an article published in the newspaper and code it according to the parameters found below in an accurate way. That is, you have to go parameter by parameter and check whether it exists in the article or not*.

*If the parameter is not met at all, the score should be 0*.

*If the parameter is met, the score should be 1*.

*Score each question in a step-by-step way to be sure you arrive at the right answer*.

*These are the parameters:*

1. *Is there any mention of suicide in the headline?*
2. *Does the article appear on the front page?*
3. *Was the suicidal person described as a celebrity?*
4. *Does the article report a single reason for the suicide or suicidal behavior?*
5. *Does the article insinuate a connection between a specific life event and suicide or suicidal behavior?*
6. *Does the article insinuate a connection between social status (any status or social role; it does not have to be a high status; e.g., soldier, celebrity, doctor, student, etc.) and suicide or suicidal behavior?*
7. *Does the article insinuate a connection between mental state and suicide or suicidal behavior?*
8. *Does the article include inappropriate images?*
9. *Does the article present any myths regarding suicide or suicidal behavior?*
10. *Does the story include glorifying descriptions of the suicide or suicidal behavior?*
11. *Is the suicide or suicidal behavior’s method described in detail (e.g., a description of the shooting, hanging, jumping)?*
12. *Is the story descriptive of the location of the suicide or suicidal behavior?*
13. *Does the story not inform the reader about suicide warning signs/risk factors?*
14. *Does the story not include any information about prevention?*
15. *Does the story not include any information about intervention?*

*Remember to work step by step for each question*.

While the prompt was initially developed for ChatGPT-4, we applied the same prompt to Claude AI to assess cross-model performance and generalizability. This approach was chosen to ensure consistency across rapidly evolving AI models and to explore the potential for wider implementation. The focus on ChatGPT-4 was partly due to its capability to be packaged as a custom GPT, facilitating potential future distribution to end-users.

### Procedure

Two psychology students, one pursuing a B.A. and the other an M.A., independently evaluated each of the 40 articles according to the screening criteria. This dual-assessment approach was employed to enhance the reliability of the data through inter-rater agreement. Following the manual evaluation, all 40 articles were processed through two LLMs, ChatGPT-4 and Claude.AI, to document their respective assessments. This procedure was designed to compare the analytical capabilities of the LLMs against the human-coded data, thereby enabling an examination of the efficacy and consistency of automated text analysis in the context of psychological research on suicide reporting.

### Statistical analysis

In order to answer the first RQ and to assess the level of agreement between the two human evaluators and the two AI-based systems, an intraclass correlation coefficient (ICC) was employed. ICC values range from 0 to 1, where 0 indicates no reliability and 1 indicates perfect reliability. Values less than 0.5 are generally considered to indicate poor agreement. Values between 0.5 and 0.75 indicate moderate agreement. Values between 0.75 and 0.9 indicate good agreement, and values greater than 0.9 indicate excellent agreement^35^.

Afterwards, the questionnaire’s items were divided into 4 dimensions: prominence, complexity, sensationalism, and prevention^32^. To assess the level of agreement between the two human evaluators and the two AI-based systems, a Fleiss’ kappa analysis, which is suitable for assessing inter-rater agreement for ordinal data, was employed for each dimension^36^.

To answer the second RQ and to assess the differences between the four raters (two human raters and two AI raters) a repeated measures analysis of variance (ANOVA) and post hoc comparisons using Tukey’s honestly significant difference (HSD) were used to check whether there were differences between the raters in the overall score. The comparisons between the two human raters and the two AI-based systems in the four dimensions (Prominence, Complexity, Sensationalism and Prevention) of the questionnaire were made by a Friedman test, which is suitable for comparing four related groups on an ordinal dependent variable. The Wilcoxon test was used as a post-hoc test to reveal the differences between the raters.

### Ethical considerations

This study was exempt from ethical review since it only evaluates AI chatbots and no human participants were involved.

## Results

As stated previously, we used the ICC to evaluate the consistency and agreement among four distinct raters, comprising two human evaluators and two AI-based systems. The use of the ICC in this context is predicated on its ability to quantify the level of concordance among raters, thereby providing a robust framework for comparing the precision and reliability of human judgment against the algorithmic determinations made by AI. Table 2 presents the ICC results between pairs of raters. The ICC results presented in Table 2 indicate that there was a very high level of agreement between HumanRater1 and HumanRater2.

**Table 2:** Level of agreement between pairs of raters

	HumanRater1	HumanRater2	Claude.AI	ChatGPT-4
HumanRater1				
HumanRater2	0.92 [0.85;0.96]			
Claude.AI	0.73 [0.46;0.86]	0.78 [0.49;0.90]		
ChatGPT-4	0.81 [0.64;0.90]	0.87 [0.75;0.93]	0.68 [0.27;0.85]	

The values outside the parentheses are the ICC estimates. The values within the parentheses represent the 95% confidence interval for each ICC estimate.

The ICC results between Claude.AI and HumanRater1 suggest a good level of agreement, although with a wider confidence interval. Also, the ICC results between Claude.AI and HumanRater2 reflect good agreement, but with a substantial range in the confidence interval.

The ICC results between ChatGPT-4 and HumanRater1, and the ICC results between ChatGPT-4 and HumanRater2, indicate strong agreement.

Lastly, the ICC results between the two AI raters, Claude.AI and ChatGPT-4, suggest a moderate level of agreement and the widest confidence interval range, indicating a considerable amount of uncertainty in the estimate.

The general conclusion from the ICC results table is that there was strong agreement – indeed, the highest among all pairs – between the human raters. The agreement between human raters and AI raters was also strong, suggesting that the AI systems were relatively consistent with human judgments. However, the agreement between the two AI raters, Claude.AI and ChatGPT-4, was somewhat lower and was the lowest pairwise agreement observed in the table.

Moreover, the confidence intervals were quite wide for some of the AI comparisons, especially between the two AI systems, indicating a higher level of uncertainty in those measurements. The narrower confidence intervals between the human raters imply greater precision in the estimate of their agreement.

**Fig. 1:**
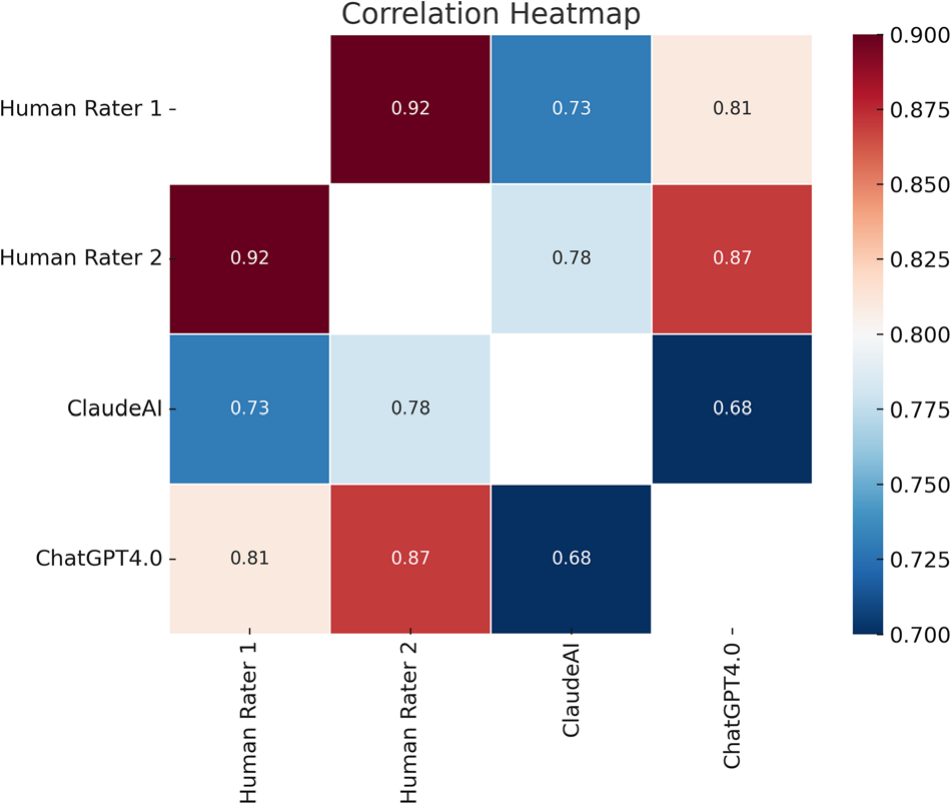
This heat map displays the Intraclass Correlation Coefficient (ICC) values between four raters evaluating suicide-related articles according to World Health Organization guidelines. The raters include two human evaluators (HumanRater1 and HumanRater2) and two artificial intelligence systems (Claude.AI and ChatGPT4.0).

Overall, although there was evidence of good reliability across both human and AI raters, the strongest concordance existed between the human raters, and there was more variability and less certainty in the agreement involving AI raters. Figure 1 represents the results using a heat map.

**Fig. 2:**
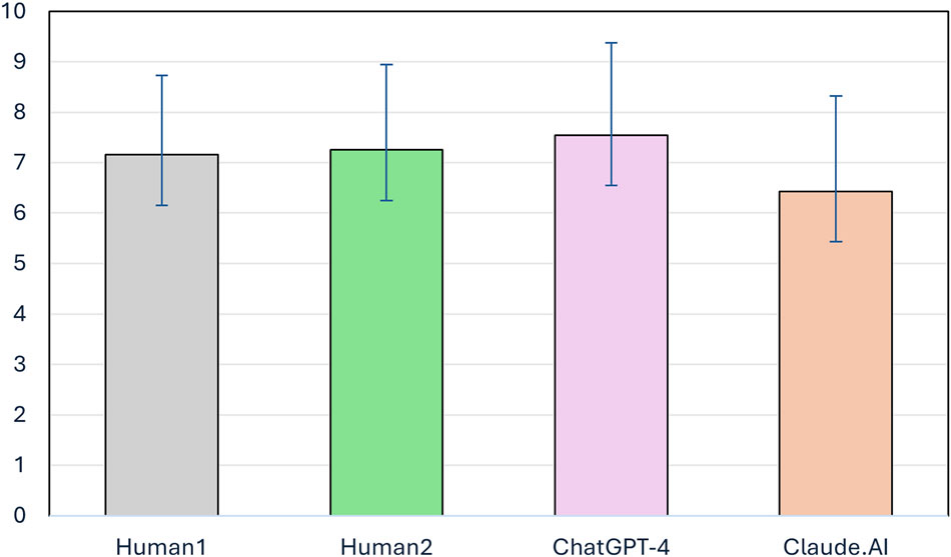
Mean compliance with WHO guidelines by human and AI raters. This bar graph displays the mean ± SEM of article compliance with World Health Organization (WHO) guidelines for reporting on suicide, as evaluated by four different raters: two human raters (Human1 and Human2) and two artificial intelligence systems (ChatGPT-4 and Claude.AI).

In order to answer RQ2 and to assess the differences between the raters in their overall estimations, a repeated measures ANOVA was conducted. Results indicated a significant effect of the variable, (F_(3, 117)_ = 9.80, *p* < 0.001). Post hoc comparisons using Tukey’s HSD indicated that the mean scores for Claude.AI were significantly lower than those for Human1 (*p* = 0.0013), Human2 (*p* = 0.0079), and ChatGPT-4 (*p* = 0.0371). No other significant differences were observed between the groups. Table 3 shows the descriptive statistics of raters’ estimations. Figure 2 shows a visual representation of the results.

**Table 3:** Descriptive statistics of raters’ estimations

Rater	Mean	SD
Human1	7.15	1.58
Human2	7.25	1.69
ChatGPT-4	7.54	1.83
Claude.AI	6.43	1.89

In order to compare between the two human raters and the two AI-based systems in each one of the four dimensions of the questionnaire (Prominence, Complexity, Sensationalism and Prevention), a Friedman test was used. The Friedman test is suitable for comparing four related groups on an ordinal dependent variable. Table 4 shows the results of the analyses.

**Table 4:** Mean ranks and Friedman test results for ratings of prominence, complexity, sensationalism, and prevention by two human raters and two AI-based systems

	Human1	Human2	Claude.AI	ChatGPT-4	Chi-square (df=3)	Sig
Prominence	2.56	2.56	2.17	2.70	17.75	<0.001
Complexity	2.50	2.58	1.94	2.99	23.46	<0.001
Sensationalism	2.79	2.56	2.35	2.30	9.94	0.02
Prevention	2.49	2.49	2.53	2.50	0.23	0.97

Table 4 presents the Friedman test results for comparing two human raters and two AI-based systems across the four questionnaire dimensions. Significant differences were noted in prominence and complexity (*p* < 0.001), and sensationalism (*p* = 0.02), indicating variation in ratings across groups. However, no significant difference was observed in prevention (*p* = 0.97), suggesting consistent ratings among the four groups for this dimension (Prominence, Complexity, Sensationalism and Prevention).

The Wilcoxon test was used as a post-hoc test to reveal the differences between the raters. For the prominence dimension, the rating of Claude.AI was significantly lower than that of the two human raters and ChatGPT-4.

For the complexity dimension, ChatGPT-4 showed a significantly higher score than did the three other raters, and Claude.AI performed significantly lower than the rest of the raters. There were no significant differences between the two human raters. For the sensationalism dimension, Claude.AI showed a significantly lower score than the first human rater, but no other differences were found between the other raters.

## Discussion

The aim of the current study was to examine to what extent LLMs, namely ChatGPT-4 and Claude.AI, could evaluate suicide-related articles relative to the WHO recommendations, in comparison with human raters as per the 2023 study by Levi-Belz et al.^32^ Such an understanding will be an important first step toward using this technology to produce a critical follow-up of articles on suicide that are published in the press or online.

In the current study we found that the AI system analysis was compatible with the human rater analysis and was able to issue insights regarding an article’s degree of compliance with the WHO recommendations. In the case of ChatGPT-4, a combination of high ICC values with the human raters indicates sensitivity to the severity differences between the articles, whereas the absence of a significant difference in the final severity ratings suggests accurate calibration. Even in the assessment of the subscales (prominence, complexity, sensationalism, and prevention), no differences were found between ChatGPT-4 and the human raters, except for complexity, where ChatGPT-4 rated the articles more severely, meaning it was stricter in its evaluation.

A study by Levi-Belz et al.^32^ examined 200 articles about suicide and suicidal behavior published in Israel’s two highest-circulation daily newspapers between 2012 and 2019. The study found that across all time points, ~49.35% of the articles adhered to WHO guidelines for responsible reporting on suicide. Specifically, 49 articles were analyzed from 2012, 63 from 2016 to 2017, and 88 from 2018 to 2019. Considering the significant negative impact of irresponsible reporting on suicide, and the fact that attempts to change reporting habits through raising awareness or educating editors and journalists have been unsuccessful^32^, we believe that the solution presented in this article, which introduces a simple, automated process with the potential to operate in any language, could be an effective and efficient solution to the problem, with wide distribution and low cost. On a theoretical level, these findings reinforce previous studies that have demonstrated the ability of LLMs to successfully process textual content and extract insights from it in the field of mental health in general^1,2,6,37,38^ and specifically on the topic of suicide^5,8,39^. One study showed that ChatGPT-4 assessed the risk of suicidal behavior identically to the way in which mental health professionals assessed it in four different case descriptions, demonstrating its potential to extract insights with regard to suicide specifically^8^. Regarding Claude.AI, it is evident that its performance was slightly inferior to that of ChatGPT-4. The difference may be attributed to the fact that the original prompt was designed in GPT’s interface and tailored to it. It is conceivable that creating a prompt specifically for Claude.AI could have enhanced its performance.

Our study highlights potential risks associated with AI-based screening of suicide-related articles. False positives could undermine the system’s credibility, potentially leading to journalist resistance and reduced willingness to use the tool, while false negatives might allow problematic content to be published, inadvertently reinforcing harmful reporting practices. To mitigate these risks, we propose a human-in-the-loop approach as an initial implementation stage. This hybrid model combines AI efficiency with human expertise: the AI system performs initial article screening based on WHO guidelines, followed by a trained human expert’s review of flagged content. This approach not only addresses the risk of erroneous classifications but also maintains the system’s credibility among journalists, creates a feedback loop for continuous AI improvement, and provides an opportunity for ongoing journalist education about responsible suicide reporting. As the system’s accuracy improves over time, a gradual transition towards increased automation could be considered, always retaining some level of human oversight to ensure ethical and contextually appropriate content evaluation. Additionally, future research could explore the potential of implementing this technology as a client-side filter, similar to email spam filters, which could empower readers to screen content before consumption. This alternative application could complement journalistic efforts and provide an additional layer of protection for vulnerable individuals.

The study had several limitations. Primarily, the investigation was confined to the analysis of newspaper articles, both online and in print, neglecting other forms of media such as radio and Internet news platforms. This limitation is significant given the burgeoning influence of digital and social media, necessitating a comprehensive evaluation of the implications these channels have for suicide reporting practices. Additionally, the scope of the research was limited by a relatively modest sample size, drawn from articles published in newspapers with high circulations. This selection criterion raises concerns about the representativeness of the findings, as they may predominantly reflect the media consumption habits of the broader populace rather than those of distinct demographic groups.

The assessment of the articles was subject to the interpretations of trained evaluators regarding adherence to established media guidelines, a factor that both supports and potentially limits the study’s validity and reliability. Despite the expertise of these raters, the possibility that pertinent articles were overlooked due to the restrictive nature of the keyword search cannot be discounted. Although the aim of this approach was to ascertain the influence on compliance with suicide reporting standards, it is imperative to acknowledge that the analysis was circumscribed to just two AI models—ChatGPT-4 and Claude.AI. The investigation’s breadth could be enhanced by extending the analysis to encompass a wider array of AI models and their evolution in media reporting practices over time.

Given the complexities involved in media reporting on suicide and the observed partial adherence to established guidelines, this study suggests the potential for using AI to disseminate knowledge and relevant criteria more widely. This approach has shown promise in various languages, though it requires further research and development. On a practical level, AI could offer a feasible solution for evaluating media content. By using AI to assess articles, we may be able to facilitate the correction of harmful narratives and contribute to suicide prevention efforts. The findings of this study could be a starting point for exploring the possibility of automatically identifying articles that do not meet the established criteria and quickly alerting relevant parties to assist in making corrections. Going forward, researchers could investigate the extent to which ChatGPT can propose more appropriate ways to report on suicide, potentially replacing problematic sentences in articles. This approach might help articles meet the WHO criteria more rapidly, contributing to changes in reporting practices. Additionally, AI could potentially provide a summary of guideline adherence level and offer suggestions for improvement to writers and editors. In the future, this technology could be used not only to evaluate articles but also to correct them or assist journalists in writing articles in accordance with the guidelines. The approach developed in this study could potentially be extended to detect irresponsible coverage of suicide on online platforms and social media. Moreover, it might be adapted to monitor posts by individuals at risk for suicidal behavior. Future applications could leverage advanced video analysis techniques, such as Google’s Gemini Pro 1.5, to analyze TikTok videos or other social media content. However, such applications would require further research to address the unique challenges of these mediums, including the volume and variety of content, as well as the informal nature of many social media posts.

AI could possibly play a role in news coverage related to individuals with special needs and other sensitive topics, such as homicide cases and reporting on casualties in conflict zones. Furthermore, this scenario illustrates how AI might be used to support mental health proactively, not just at an individual level but societally. Such preventive measures, facilitated by AI, could have an impact on public mental health. Nevertheless, further research is needed to fully understand its potential and limitations.

## Supplementary information

Supplementary information

## Data Availability

The data that support the findings of this study, including the original Hebrew news articles and our analyses, are available as supplementary material with this publication.
