# Supplementary material for: Applying language models for suicide prevention: evaluating news article adherence to WHO reporting guidelines
Source: Npj Ment Health Res. 2025 Jun 20;4:25. doi: 10.1038/s44184-025-00139-5 (PMC12181428; doi:10.1038/s44184-025-00139-5)
Supplement: Supplementary file 1 — Supplementary information [file 44184_2025_139_MOESM1_ESM.pdf]

Article.num	Human_1	Human_2	Mean_Hun	Claude	GPT	links
1	8	8	8	8	8	https://www.israelhayom.co.il-military-life/world-news-military/life/article13597041
2	5	5	5	6	6	https://www.israelhayom.co.ilarticle/776999
3	8	8	8	7	10	https://ww.w.ynet.co/il/articles-L,0,7340,4795523html.00
4	9	8	8	8	8	https://ww.w.ynet.co/il/articles-L,0,7340,4463259html.00
5	6	3	6	2	4	https://ww.w.ynet.coil/health/article/s18uizxah
6	9	9	9	6	10	https://ww.w.ynet.co/il/articles-L,0,7340,5247667html.00
7	10	10	10	9	9	https://ww.w.ynet.co/il/articles-L,0,7340,3764525html.00
8	7	7	7	6	7	https://www.israelhayom.co.ilarticle/631715

9	7	7	7	5	6	https://www.w.ynet.co/il/articles-L,0,7340,5666099html.00
10	7	7	7	6	8	https://www.israelha/yom.co.il sport/worl-d soccer/article/14793882
11	9	9	9	7	7	https://www.israelha/yom.co.il article/674203
12	9	10	8	9	7	https://www.israelha/yom.co.il article/670929
13	6	6	6	4	8	https://www.w.ynet.co il/news/article/sycd0dtv2
14	5	6	5	4	5	https://www.w.ynet.co il/news/article/byyxtrsrk
15	4	4	4	3	2	https://www.w.ynet.co il/health/article/sy2ipepfp#autoplay
16	7	6	7	8	6	https://www.w.ynet.co il/news/article/b1b6pe9ky
17	5	5	5	3	5	https://www.w.ynet.co/il/articles-L,0,7340,4851053html.00

18	3	3	3	2	4	https://www.ynet.co.il/articles-L,0,7340,4847660.html.00
19	7	8	7	7	8	https://www.israelhayom.co.il/article/763633
20	6	6	6	5	7	https://www.israelhayom.co.il/article/772403
21	5	7	5	6	7	https://www.israelhayom.co.il/article/739529
22	5	8	5	9	9	https://www.israelhayom.co.il/article/532705
23	9	9	9	6	9	https://www.israelhayom.co.il/article/733883
24	7	7	7	7	8	https://www.israelhayom.co.il/article/45322
25	7	7	7	8	7	https://www.israelhayom.co.il/article/242813
26	9	10	9	5	10	https://www.israelhayom.co.il/news/world-news/other/article/14569419

27	7	7	7	7	8	https://www.israelhayom.co.il/article/780327
28	8	8	8	6	8	https://www.israelhayom.co.il/article/600411
29	7	7	7	8	8	https://www.israelhayom.co.il/article/78391
30	9	9	9	7	9	https://www.israelhayom.co.il/article/401553
31	8	9	8	8	9	https://www.israelhayom.co.il/article/86437
32	8	8	8	9	9	https://www.israelhayom.co.il/article/469617
33	7	7	7	7	8	https://www.israelhayom.co.il/article/506129
34	7	8	7	6	8	https://www.israelhayom.co.il/news/world-news/europe/article14919685
35	6	6	6	6	7	https://www.israelhayom.co.il/article/615991

36	7	7	7	5	8	https://www.israelhayom.co.il/article/578683
37	8	8	8	8	9	https://www.israelhayom.co.il/article/305743
38	8	7	7	7	11	https://www.israelhayom.co.il/article/85501
39	9	9	9	9	10	https://www.israelhayom.co.il/article/35074
40	8	7	8	8	6	https://www.israelhayom.co.il/article/674651
